# Examination of Knowledge and Fear Levels of Breast Cancer With the Spiritual Characteristics of Nurses

**DOI:** 10.3389/fpubh.2018.00331

**Published:** 2018-11-20

**Authors:** Ayse Cal, Mukerrem Kabatas Yildiz, Ilknur Aydin Avci

**Affiliations:** Department of Public Health Nursing, Faculty of Health Sciences, Ondokuz Mayıs University, Samsun, Turkey

**Keywords:** breast cancer, spirituality, nursing, knowledge, fear, coping behaviors

## Abstract

**Introduction:** Breast cancer is a common problem and it is important to understand the beliefs that increase awareness of breast cancer and guide early diagnosis behaviors. This research is planned to examine the knowledge and fear levels of breast cancer along with the spiritual characteristics of nurses.

**Methods:** This is a descriptive type research. The domain of the research consists of women nurses working in Health Sciences University Samsun Education and Research Hospital. Sampling was not undertaken, rather 327 nurses who were on duty between January and May of 2016 and were willing to cooperate were incorporated into the study. Data was collected by use of the survey forms, “Breast Cancer Fear Scale” and “Comprehensive Breast Cancer Knowledge Test (GKMBT).” Data were analyzed with SPSS 21 Software.

**Results:** Nurses who participated in the research had an average of age of 32.27 ± 1.04 years, 60.6% of whom were married, and had an average duration of nursing practice of 12.49 ± 9.92. The nurses' breast cancer fear level point average was 26.11 ± 6.58, the GMKBT scale general information sub-dimension point average was 7.20 ± 2.81, the treatability sub-dimension point average was 5.80 ± 1.68, and the total point average was found out to be 12.87 ± 2.81. It was determined that nurses' knowledge levels of breast cancer were not related to fear levels.

**Conclusion:** It was concluded that the nurses' breast cancer fear level was high and their knowledge level was moderate. In line with the results obtained, it might be recommended that studies should be made to increase the nurses' knowledge and awareness on breast cancer.

## Introduction

Breast cancer is an important public health problem that affects women's health worldwide (1). It is the most common type of cancer among women (29%) and is the second leading cause of cancer death (14%) (2). According to the cancer rates in Turkey, breast cancer is first among the top ten types of cancer seen in women (45.9 out of 100,000) (3).

The incidence of breast cancer increases daily. It is the type of cancer with the highest survival rate (41%) and at the same time it can achieve highly successful treatment results at early diagnosis and can reduce the side effects related to the disease (2, 4). For this reason, it is important to understand the beliefs that increase awareness of breast cancer and guide early diagnosis behaviors.

There is a significant effect of community culture on the attainment of health behaviors. The subjective societal perception of health may be decisive on health behaviors. Regarding whether someone accepts a health issue as a problem, it is an important influence on the fight against that health problem. Studies show that longer-term work should be done to prevent problems that society does not consider to be a health problem (5, 6). The culture of a society is a harmony of life, belief, practices, and habits. Protective health behaviors are closely related to community culture as they are applications aimed at healthy people.

Cultural and religious factors need to be understood in order to understand the relationship between health and disease in societies (5). The beliefs and attitudes of individuals on the concept of health and illness are shaped by the culture in which they live. In the direction of beliefs, people's approaches to health and illness and their healing patterns also change. In this context, it is useful to understand beliefs that are effective in leading positive behaviors (6). Health personnel should reveal unique beliefs of people that they care for and in what way these beliefs affect the health of people (5).

As in all other religions, beliefs in Muslim Turkish society also affect every period of life and health behaviors. The faith in fate has an important place in Islamic societies. Destiny of faith means that Almighty Allah knows and appreciates the time and place of everything as infinite as forever. Here, the prevailing view, “What should I do, that is Allah's blessing, it is my destiny,” is widespread (5). The study of Polek and Hardie examining the fateful beliefs of Asian-Americans emphasizes that fateism is a prevailing belief and that cancer is perceived as fate in these societies. It has been shown that fatalism is an important influence on the cancer screening behaviors of societies and may lead to a delay in the early diagnosis and treatment of cancer (7). Baider and Goldzweig have shown that Muslims interpret the pain as atonement for their sins in their study of a Muslim woman with breast cancer in Israel, and that this interpretation helped patients and their families to cope with the diseases (8). Culture and religion have positive effects on health as well as negative effects (5). Although nurses do not participate in the opinions of community members to whom they give health service, they should evaluate the event independently of their own individual values, with the view of that person (5). Accepting practices that are contradictory to the culture of the community is difficult and sometimes impossible. One of the important factors in the perception of fate is fear. Since people are afraid of the diseases they have attributed to death, it can be thought that they leave the course of their disease to fate.

Nowadays thoughts of cancer cause fear in most people. When breast cancer is diagnosed early, the life span of most people can be prolonged by developing diagnostic and treatment methods. For this reason, one of the most important steps to be taken is to ensure that every society poses breast cancer risks in itself, identifies risk groups and promotes screening programs (9).

All health personnel must know the cultures and religious beliefs of the community in order to be better able to care for and treat the community in which they live and serve. Nurses are seen as an important source within the health industry in improving breast cancer knowledge and attitudes (10). Nurses undertaking community health care also constitute an important target group in terms of determining the level of knowledge since they are role models for gathering health behaviors. At the same time, eliciting the subjective perceptions of health and disease, getting information about culture by health personnel who will provide service to the community will affect the quality and acceptability of the health care that the health staff will present. Nurses spend a lot of time in the hospital environment, and nurses' role expansion and specialization are becoming more complex. Nurses are core factors of various health problems and disease development. Alternating shift schedules can lead to a risk of cancer (11). Thus, the longer the shift period, including long-term night shifts, the greater the risk of breast cancer. In the globalizing world, international migration results in many cultures living together. For this reason, it is inevitable for nurses to reflect more on their practice of providing multicultural care in the framework of holistic care. In this respect, this research was planned to examine the spiritual characteristics of nurses and the level of knowledge and fear of breast cancer.

## Materials and methods

### Location and time of the study

The research was carried out in a university and research hospital in Samsun between January and May 2016 as a descriptive research.

### Sample of research

The setting of the research was formed by (501) nurses working in an education and research hospital in Samsun. Sampling was not undertaken for the survey as the entire nursing staff was included in the research. Among the dates on which the research was conducted, 327 women who were not on leave and who agreed to participate in the survey formed the surveyed population of nurses. 65.27% of the total population of nurses was reached in the study. The dependent variables of the study were nurses' comprehensive breast cancer knowledge test scale scores and breast cancer fear scale scores; independent variables were variables related to age, education, gender, years of work, service, and fate perception of nurses.

### Data collection tools

In the questionnaire form, there were 20 questions developed by the researchers in light of the related literature. This was then presented to the nurses,including questions about breast health and fate perception in addition to the sociodemographic characteristics of the nurses.

The Breast Cancer Fear Scale was developed in 2004 by Victoria Champion et al. (12) and its translation into Turkish was made by Seçginli (13). The Cronbach Alpha coefficient of the scale is 91. The scale used to determine the relationship between breast cancer, mammography behavior and emotional reactions of women is composed of eight items, which are evaluated with the following expressions: (1) I strongly do not agree, (2) I do not agree, (3) Undecided, (4) Partially agree, (5) I agree. The highest score to be taken from the scale is 40 and the lowest score is 8. In assessing the scores from the breast cancer fear scale, low-level fear would be between 8 and 15 points, middle-level fear between 16 and 23 points, and high-level fear between 24 and 40 points. The Cronbach Alpha coefficient for this study was 0.88.

The Comprehensive Breast Cancer Knowledge Test (CBCK) It was developed by Stager in 1993 (14). A Turkish version of the test was made by Basak in 2015 (15). For the entire test, the Cronbach Alpha number was found to be 0.90. The total of 20 items in the test are answered as true-false. There are 8 correct (3, 4, 7–10, 13, 16) and 12 incorrect (1, 2, 5, 6, 11, 12, 14, 15, 17–20) statements in the questions. There were two dimensions in CBCK, general knowledge and treatability. Questions ranging from 1 to 12 are in relation to general information on breast cancer, while those from question 13 to question 20 contain information on the treatability of breast cancer. After the test application has been performed, according to the answer key of the scale, the “correct” answers were assigned a 1, while “wrong” and blank answers were given a score of 0 (14). The Cronbach Alpha coefficient for these data collection forms was 0.50.

### Collection of data

The purpose of the research was explained to all nurses in person. The nurses self-administered the 20-itm questionnaire, “Breast Cancer Fear Scale,” ad well as the “Comprehensive Breast Cancer Knowledge Test” to generate the data. The data collection form was evaluated by pre-application in a group of 10 people who have features of sample group all forms were applied without any correction in the pre-application result. The forms were completed in-person by the nurses and the data collection period lasted 15–20 minutes.

### Evaluation of data

The percentage values, mean, and standard deviation were obtained by using descriptive statistics with the aid of IBM SPSS 21.0. The Kruskal-Wallis test, the Mann-Whitney *U* test, and correlation analysis were used to define the difference between dependent and independent variables.

### Research ethics

For the study, written consent was obtained from the university's non-invasive clinical research ethics committee (no. 2015/358) and from the head of the hospital where the study was conducted. Participants were informed that the results would be used scientifically, they had the opportunity to leave the study at will as voluntary participation was essential, and their identity would remain hidden. Participants were approved as participant-volunteers in accordance with the principles of the Declaration of Helsinki (https://www.wma.net/policies-post/wma-declaration-of-helsinki-ethical-principles-for-medical-research-involving-human-subjects/, 2016) by giving information about the research verbally to the nurses (16).

## Results

The mean age of the nurses participating in the study was 32.27 ± 1.04 (min: 18, max: 58), 60.6% were married, and the duration of nursing practice was 12.49 ± 9.92 (min: 1, max: 39). 19.6% had breast cancer stories in their family, 50.0% performed BSE every month, 34.3% had a clinical breast examination, and 20.5% had a mammography examination 25.7% of the nurses had previously had breast cancer patients, 22.6% of the breast cancer knowledge levels were adequate, and 60.6% of the breast cancer knowledge levels were moderate. Together with this, 84.1% of the nurses said that they wanted to participate in training on breast cancer. 17.7% of the nurses who participated in the study had previous problems with breast health, 35.5% of them answered “I may be at any period of my life,” 30.3% answered “I may be if it is in my destiny,” and 26.0% answered “I do not want to think” about being breast cancer in Table 1.

**Table 1:** Nurses' socio-demographics, knowledge and practices related to breast health.

**Features**	$\bar{\text{X}}\;\pm$**SS**	
Age	32.27 ± 1.04	
Duration of nursing practice	12.49 ± 9.92	
	***n***	**%**
**Marital status**		
Married	198	60.6
Single	129	39.4
**Breast cancer story**		
Yes	64	19.6
No	263	80.4
**Making Breast Self-Exam (BSE)**		
Yes	200	61.2
No	127	38.8
**Frequency of making BSE**		
Monthly	100	50.0
Once every three months	45	22.5
Once every six months	33	16.5
Yearly	22	11.0
**Clinical Breast Exam (CBE)**		
Yes	112	34.3
No	215	65.7
**Mammography**		
Yes	67	20.5
No	260	79.5
**Caring for breast cancer patients**		
Yes	84	25.7
No	243	74.3
**Information about breast cancer**		
Adequate	74	22.6
Average	198	60.6
Inadequate	55	16.8
**Request breast cancer education**		
Yes	275	84.1
No	52	15.9
**Having trouble with breast health**		
Yes	58	17.7
No	269	82.3
**The thought of having breast cancer**		
I might in a period of my life	116	35.5
I might if it is in my destiny	99	30.3
I do not want to think about it	85	26.0
I have no idea	27	8.3

When nurses were questioned about their attitudes at the time of the disaster, 59.6% chose the expression “Things affect me very much but I try not to lose myself completely, I endure.” 93.6% of the nurses stated that they gave importance to spiritual values, and 52.6% said that diseases strengthened their spiritual beliefs.

Breast cancer fear levels differ according to nurses' attitudes at the time of disaster (*p* < 0.05). Postdoc evaluation of the statistical significance was found to be originated between two options: “Things affect me very much, I feel devastated” and “I quickly overcome the negative effects of events and continue to work for my goals” (*p* < 0.05).

There was no difference between the levels of fear of breast cancer, the nurses that gave importance to spiritual values, and thoughts about influence of disease for spiritual belief (*p* > 0.05) in Table 2.

**Table 2:** Attitudes of nurses to some spiritual characteristics and Breast Cancer Fear Scale averages distribution.

	***n***	**%**	**Median (min-max)**	**Mean rank**	**Statistics**
**Attitudes in the process of disasters**					
Things affect me very much, my world gets dark, all my wishes and desires are destroyed, and I feel devastated.	34	10.4	29.50 (10–39)^a^	209.31	KW = 9.035
					*p* < 0.05
Things affect me very much but I try not to lose myself completely, I endure.	195	59.6	27.00 (8–40)^b^	156.66
I quickly overcome the negative effects of events and continue to work for my goals.	98	30.0	27.00 (8–40)^bc^	162.89
**Situation of caring on spiritual values**					
Care	306	93.6	27.00 (8–40)	164.74	U = 2987.00
Don't care	21	6.4	27.00 (8–40)	153.24	*p* > 0.05
**Thoughts about influence of disease for spiritual belief**					
The disease strengthens my spiritual belief.	172	52.6	27.00 (8–40)	164.04	KW = 0.793
The disease does not affect my spiritual belief.	20	6.1	28.00 (8–39)	177.35	*p*>0.05
I have no idea.	136	41.3	27.00 (8–40)	161.97

a-c: *There is no difference between the groups with the same letter*.

Nurses participating in the study said that they preferred spiritual coping methods for dealing with the diseases; 92.7% of them preferred praying, 55.4% preferred reading Quran, 55.4% preferred performing a prayer Alternate coping methods were also present, as 41.3% preferred traveling, 31.2 preferred reading books and newspapers, and 16.5% prefered doing handcraft in Table 3.

**Table 3:** Spiritual and alternative coping strategies with diseases for nurses.

**Methods of coping**	**Uses**		**Doesn't use**	
	***n***	**%**	***n***	**%**
Praying	303	92.7	24	7.3
Reading Quran	181	55.4	146	44.6
Performing Prayer	178	54.4	149	45.6
Visiting shrine/entombed saint	23	7.0	304	93.0
Taking a vow	47	14.4	280	85.6
Getting an amulet	10	3.1	317	96.9
Reading book or newspaper	102	31.2	225	68.8
Writing a poem	10	3.1	217	96.9
Doing handcrafts	54	16.5	273	83.5
Traveling	135	41.3	192	58.7
Smoking cigarettes	46	14.1	281	85.9

^*^The participant have marked as many options as they want. All responses were based on nurses' self-report.

It was found that nurses' breast cancer fear levels were higher but breast cancer knowledge levels were moderate in total score, general knowledge, and treatability subscales. No relationship between breast cancer fear levels and breast cancer knowledge of nurses was found (*r* = 0.001) (*p* > 0.05). This was also true regarding the relationship between breast cancer fear levels and duration of nursing practice (*r* = 0.036) (*p* > 0.05).

Breast cancer knowledge levels of nurses who performed BSE every month were significantly higher than the others (KW = 12.359) (*p* < 0.05).

When the attitudes of nurses in the presence of illnesses are questioned, the levels of breast cancer fear of those who say “diseases affect me much, my world becomes upside-down, I feel devastated” and “I influenced a lot but I try to be patient” has been found to be significantly higher than those of the words “I get over the negative effect of diseases shortly and it does not affect me” (KW = 9.035) (*p* < 0.05) in Table 4.

**Table 4:** Nurses' breast cancer fear scale and comprehensive breast cancer knowledge test score averages distribution.

	**Average**	**S.D**	**Min**.	**Max**.	**Cronbach α**
**Breast cancer fear scale**						
Total	26.11	6.58	8	40	0.88	*r* = 0.001^a^ *p* > 0.05
**Comprehensive breast cancer knowledge test**						
General information	7.20	2.81	0	12	0.30
Treatability	5.80	1.68	0	8	0.54
Total	12.87	2.81	0	19	0.50
**Frequency of making BSE**	**Median**		**Min**.	**Max**.	**KW**	***p***
Monthly	14.00		0	18	12.359^b^	< 0.05
Once in three months	13.00		7	18	
Once in six months	12.00		0	17	
Yearly	13.00		6	19	
**Attitudes in the process of disasters**						
Things affect me very much, my world gets dark, all my wishes and desires are destroyed, and I feel devastated.	29.50		10	39	9.035^c^	< 0.05
Things affect me very much but I try not to lose myself completely, I endure.	27.00		8	40	
I quickly overcome the negative effects of events and continue to work for my goals.	27.00		8	40	

a *Correlate of breast cancer fear levels and breast cancer knowledge*,

b *Analysis of making BSE with breast cancer knowledge levels*,

c *Analysis of attitudes in the process of disasters with levels of breast cancer fear*.

## Discussion

In the study, it was seen that religious practices such as praying, performing a prayer, and reading Quran were the first of the nurses' spiritual coping methods. Otherwise they preferred to use alternative applications such as traveling, reading books and newspapers as coping methods. As a result of a meta-analysis of religion and spirituality in relation to cancer, it was emphasized that these variables positively affected the mental health of individuals (17). The National Cancer Institute emphasized the effect of spirituality on the lives of individuals with a patient statement “I can't handle everything on my own. But it's real hard when I have my down times. And yet, most of the time, my faith gives me strength and some sense of peace.” (18). Studies have shown that religion and spirituality influence individuals' perceptions of health and that these variables are important for patient-centered care (17, 19). The National Comprehensive Cancer Network (NCCN) guidelines recommend that cancer patients be monitored periodically to support their religious and spiritual needs (20).

In this study, 71.9% of the nurses were found to have a high level of breast cancer fear. In the studies conducted, the breast cancer fear levels of women in the general population are mostly examined and the results are different. Lee found that women's breast cancer fears were moderate at 55.6% (21). In another study, it was determined that women's perception of breast cancer as a painful and lethal disease lay on the basis of fear of breast cancer (22). In a study conducted in Turkey, women's breast cancer fear levels were found to be 85.1% (23).

The breast cancer fear levels of the nurses participating in the study were high, whereas the levels of early diagnosis behaviors were relatively low in BSE (50%), CBE (34.3%), and mammography (20.5%). Conducted studies have shown that fear of breast cancer negatively affects realizing the early diagnosis behavior in women (24–27). Koc and Salam stated that unlike the results of the study, 51.4% of females performed BSE resulting from a fear of receiving a breast cancer diagnosis (28). Contrary to the studies, there was no relationship between nurses' breast cancer fear levels and early diagnosis behaviors in this study.

It was observed that the attitudes of nurses during the process of disaster were related to the level of breast cancer fear. The increase in the level of breast cancer fear negatively affected the attitudes of individuals in the moment of disaster. In the study, the attitudes of the nurses at the time of the disaster were questioned and 59.6% of them chose the phrase “Things affect me very much but I try not to lose myself completely, I endure.” Here, it was thought that the psychological factor underlying the patience that individuals show was the sense of destiny, and it was seen that the perception of destiny positively influenced their ability to cope with the adverse situations encountered. In addition, it is known that in Muslim communities where ideas of fate are prevalent, preventive health care for early diagnosis is very low, and individuals are encouraged to be spiritually strong and a submissive understanding of destiny prevails against diseases (29). Ersin and Bahar emphasized the existence of a fatalistic approach among the obstacles that affect the practice of individual breast cancer screening behaviors (4).

Gangane et al. (30) found that women's knowledge of breast cancer was very low. In the same study, women's living in rural or urban areas did not affect breast cancer knowledge levels (30). Women's breast cancer knowledge levels have been reported to be associated with the highest CSE practice and less breast cancer symptoms (31). In another conducted study, it was found that women with high levels of education had a significantly higher level of breast cancer knowledge (32). Primary reasons for not seeking an early diagnosis for breast cancer were that women were found to have a lack of knowledge (72.1%), followed by forgetfulness (12.3%), fear of finding mass (8.8%), not finding it necessary (5.2%), and finding no time (2.5%). Akhtari-Zavare et al. found that women with high breast cancer knowledge levels had also higher levels of breast self-examination (31). According to a study done in Turkey, 79.2% of women feel they have a deficiency of information about CSE, 62% of them about risk factors, and 52% of them about breast cancer symptoms (33).

In conclusion, it was found that nurses had a high level of breast cancer fear and a low level of knowledge. It was determined that nurses' knowledge levels of breast cancer and their breast cancer fear levels were not related. It was also found that breast cancer fear levels differ according to nurses' attitudes at the time of disaster. In view of the results obtained, it is suggested that breast cancer knowledge levels should be supported with in-service training in the context of lifelong learning in order for nurses to take an active role in the prevention of breast cancer. In addition, it is suggested to perform educational studies in which the individual's spiritual characteristics that are effective in applying early diagnosis behaviors of breast cancer are examined in depth.

## Limitations

Findings obtained from this research are confined to women nurses who work in a university and research hospital setting, who agreed to participate in the research, and as such the findings cannot necessarily be generalized to nurses working in other hospitals.

## Author contributions

IA, AC, and MK: concept, design, and funding. IA and AC: writing, analysis, and/or interpretation. AC and MK: literature review and data collection and/or processing. IA: critical review and supervision.

### Conflict of interest statement

The authors declare that the research was conducted in the absence of any commercial or financial relationships that could be construed as a potential conflict of interest.
